# Vitamin D status in diabetic Egyptian children and adolescents: a case–control study

**DOI:** 10.1186/1824-7288-39-73

**Published:** 2013-11-15

**Authors:** Seham FA Azab, Safaa Hamdy Saleh, Wafaa F Elsaeed, Sanaa M Abdelsalam, Alshaymaa Ahmed Ali, Asmaa MH Esh

**Affiliations:** 1 Department of Pediatrics, Faculty of Medicine, Zagazig University, 18 Omar Bin Elkhattab St, Al Qawmia, Zagazig City, Sharkia Governorate, Egypt; 2 Clinical Pathology, Faculty of Medicine, Zagazig University, Zagazig City, Egypt

**Keywords:** Vitamin D, Parathyroid hormone, Diabetic, Children

## Abstract

**Background:**

Recently, studies suggesting that vitamin D deficiency correlates with the severity and frequency of Type 1 (insulin-dependent) diabetes mellitus (T1DM) and that vitamin D supplementation reduces the risk of developing T1DM have been reported.

**Objective:**

In this study, we aimed to assess vitamin D status in Egyptian children and adolescents with T1DM.

**Methods:**

This was a case–control study including 80 T1DM diagnosed cases aged 6 to 16 years and 40 healthy children with comparable age and gender as the control group. For all subjects, serum 25 (OH) D levels were measured by ELISA, Serum parathyroid hormone (PTH) and serum insulin were measured by an electrochemiluminesce immunoassay. Serum glucose, Glycosylated hemoglobin (HbA1c) levels and homeostasis model assessment of insulin resistance (HOMA-IR) were also assessed.

**Results:**

Compared to the control group, serum vitamin D levels were not significantly lower in diabetic subjects (24.7 ± 5.6 vs 26.5 ± 4.8 ng/ml; *P* > 0.05). Among diabetic cases 44(55%) were vitamin D deficient; meanwhile 36(45%) cases had normal vitamin D level (*P* < 0.01). In addition, 26(32.5%) diabetic cases had 2ry hyperparathyroidism and 54(67.5%) cases had normal parathyroid hormone level; meanwhile, none of the control group had 2ry hyperparathyroidism (*P* < 0.01). Furthermore, we found a significant difference between vitamin D deficient diabetic cases and those with normal vitamin D level as regards HOMA-IR and diabetes duration (*P* < 0.01).

**Conclusion:**

Public health message on the importance of vitamin D status; especially in diabetic children and adolescents, should be disseminated to the public.

## Background

Type 1 (insulin-dependent) diabetes mellitus (T1DM) is an auto immune disease that results in the destruction of beta cells in the pancreas as a result of interactions between different susceptibility genes and environmental exposures [1].

Peak incidence occurs around puberty, and the disease is usually diagnosed before age 30. It is estimated that, by the time the DM1 is diagnosed, 80–90% of the beta cells are lost [2].

Recent studies suggest that vitamin D deficiency may increase the risk of developing autoimmune diseases including type 1 diabetes (T1DM) [3,4]. This occurs partly through loss of vitamin D modulation of the immune and inflammatory reaction in diabetes [5].

At the level of the immune system, 1, 25(OH)_2_ D_3_ inhibits the differentiation and maturation of dendritic cells and promotes their apoptosis [6], thus preventing their transformation into antigen presenting cells which is the first step in the initiation of an immune response [7]. At the level of the pancreatic islets, 1,25(OH)_2_D_3_ decreased in vivo and in vitro proinflammatory chemokine and cytokine expression (e.g., IL6), which are implicated in the pathogenesis of T1DM making *β*-cells less chemoattractive and less prone to inflammation; this results in decreased T cell recruitment and infiltration, increased regulatory cells, and arrest of the autoimmune process [8].

Studies from different countries have shown a highly variable prevalence of vitamin D deficiency ranging from 15 to 60% among children and adolescents with T1DM [9,10].

Before conducting a clinical trial of supplementing patients with T1DM, it is required to assess the existing status. In this study, we aimed to estimate vitamin D status in Egyptian children and adolescents with T1DM.

## Methods

This was a case control study performed in Zagazig University hospital from January to December 2012. The study included 80 children and adolescents diagnosed as T1DM cases (34 males and 46 females) while attending Pediatric outpatient endocrine clinic in Zagazig University Hospital. The age of the patients ranged from 6 to 16 years (mean, 11.4 years). Forty healthy children of comparable age and gender served as a control group. Subjects were excluded from the study if they had consumed cholecalciferol, calcium, multi-vitamin or mineral supplementation, vitamin D-fortified foods during the previous 3 months, or any medical co-morbidities or any other major chronic disease.

All patients and controls included were subjected to proper history taking, thorough clinical examination. Including anthropometric measurements (weight, height and BMI). Body Mass Index (BMI) was calculated as follows: BMI = weight (kg)/height (m)^2^.

In all participants, and patients with BMI of more than 30 were considered obese according to the World Health Organization classification [11]. Routine biochemical investigations included serum calcium, phosphorus, alkaline phosphatase (ALP), albumin, urea, and creatinine.

### Abnormal glucose homoeostasis

Criteria were defined under modified WHO criteria adapted for children [12]. The impaired glucose tolerance value is 2-h blood glucose (2 h. BG) _140 (7.8 mmol/l) and <200 mg/dl (11.1 mmol/l) for OGTT, and _110 mg/dl and <126 mg/dl for fasting glucose. For defined diabetes, the value is 2 h. BG >200 mg/dl for OGTT and/or >126 mg/dl for fasting glucose [12].

Homeostasis model for assessment of insulin resistance (HOMA-IR) is a convenient and beneficial method for evaluating insulin resistance, especially in obese subjects, and reflects insulin resistance obtained by euglycaemic clamp more accurately than fasting plasma insulin levels alone. It was calculated with the following formula (HOMA-IR) = [fasting insulin (uU/ml) × fasting glucose (mmol/L)] / 22.5. Insulin resistance is defined as the levels of the homeostasis model assessment for insulin resistance (HOMA-IR) greater than 3.16 [13,14].

### Estimation of serum 25 (OH) D levels

Samples for serum vitamin D level estimation were collected in heparinized amber colored glass vials to prevent photo degradation. Serum was extracted after centrifugation and then stored at–20°C until analyzed. In those who presented with ketoacidosis, samples were taken 4–5 days after normalization of pH.

Serum 25(OH)D level was measured by 25(OH) vitamin D ELISA assay kit (Eagle Biosciences, Inc.USA) according to the manufacturer’s instructions, to each well it was added 200 μl of prediluted serum sample, control or standard separately and incubated for 2 hours at 25°C, after washing, 100 μl of Enzyme Conjugate was added into each well and incubate for 30 min at room temperature, then, after washing, 100 μl of chromogen/ substrate solution was added into each well and incubated for 15 minutes at room temperature without shaking (protected from direct sunlight). Finally, 100 μl of stop solution was added to each well, and absorbance was read at 450 nm.

Vitamin D status was classified according to the American Academy of Pediatrics (AAP)/LWEPS’s recommendations on cut-off levels for states of vitamin D. A 25(OH)D level of <5 ng/mL (<12.5 nmol/L) was considered as severe deficiency, a level between 5 and 15 ng/mL (12.5-37.5 nmol/L) as deficiency, a level of 15–20 ng/mL (37.5-50 nmol/L) as insufficiency, and a level of 20–100 ng/mL (50–250 nmol/L) as normal (sufficient) [15]. Our patients were also divided into 2 groups according to their vitamin D status. Those with serum 25(OH) D levels <20 ng/mL were grouped as vitamin D insufficient and deficient, and those with serum 25(OH) D levels >20 ng/mL as normal with regard to vitamin D status [15].

Serum level of ALP and fasting blood glucose level were measured using a Dimension XL auto analyzer (Siemens, German).

### Estimation of serum PTH and serum insulin level

Serum parathyroid hormone (PTH) and serum insulin were measured by electrochemiluminescence immunoassay using Cobas e 411 immunoassay analyzer (Roch Diagnostics, GmbH, German). The manufacturer’s normal range for PTH was 15–65 pg/ml. Secondary hyperparathyroidism was defined as a PTH level greater than 65 pg/mL. HbA1c levels were measured by affinity chromatography test using (biosystem kit-USA) in which the affinity gel columns were used to separate glycated hemoglobin which binds to the column.

### Ethics

Informed parental consent was obtained to be eligible for enrollment into the study. The study was done according to the rules of the Local Ethics Committee of Faculty of Medicine, Zagazig University, Egypt.

### Statistical methods

SPSS version 19 was used for data analysis. The data are expressed as the mean ± SD or median (min-max) where appropriate. Test selection was based on evaluating the variables for normal distribution using the *Shapiro-Wilk* test. If the variables had a normal distribution, *Student's t-test* was used. If the variable did not have a normal distribution, the analysis was done using the *Mann–Whitney U* test. Categorical data were evaluated by *Pearson's chi-square* test. The correlations between variables were performed by *Pearson’s Correlation* test. *P* < 0.05 was considered significant.

## Results

There was no significant difference between diabetic cases and control group as regards age, gender, or BMI (*P* > 0.05), respectively (Table 1). Median disease duration in our diabetic cases was 17 months (range 3–52 months) (Table 1).

**Table 1:** Demographic data of studied subjects

	***Cases *** **(*n*** **=** ***80*)**	***Control *** **(*n*** **=** ***40*)**	***P***
**Male/female #**	34/46	24/16	> 0.05
**Age (years)**	11.4 ± 2.5	10.8 ± 2.3	> 0.05
**BMI (kg/m**^**2**^**)**	23.6 ± 5.7	21.8 ± 4.7	> 0.05
**Disease duration (months)**	17 (3–52)	-	-

BMI, body mass index.

Data are presented as mean ± SD or median (min-max).

*Student t*-*test* was used for comparisons. *P* value < 0.05 indicates a significant difference.

# Chi-square test.

**Figure 1:**
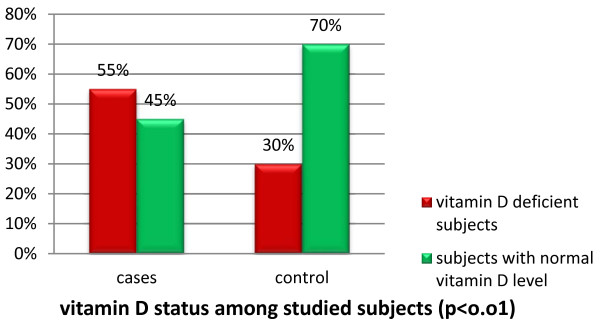
Vitamin D status among studied subjects.

Our data showed that 44(55%) diabetic cases were vitamin D deficient; meanwhile 36(45%) cases had normal vitamin D level. On the other hand, 12(30%) of the control group, were vitamin D deficient and 28(70%) had normal serum vitamin D level (*P* < 0.01; Figure 1).

**Figure 2:**
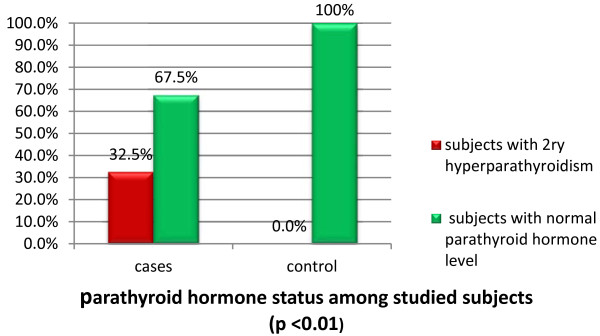
Parathyroid hormone status among studied subjects.

Of note, 26(32.5%) diabetic cases had 2ry hyperparathyroidism and 54(67.5%) cases had normal parathyroid hormone level; meanwhile, none of the control group had 2ry hyperparathyroidism (*P* < 0.01; Figure 2).

Serum calcium level was significantly lower in diabetic cases (8.5 ± 1.6 mg/dL) compared to the control group (10 ± 1.4 mg/dL; *P* < 0.01), respectively (Table 2). Meanwhile, serum phosphorus, parathyroid hormone and fasting blood glucose levels were significantly higher in diabetic cases compared to the control group (all *P* < 0.01). However, no significant difference was observed between both groups as regards to serum vitamin D level (24.7 ± 5.6 vs 26.5 ± 4.8 ng/ml; *P* > 0.05) (Table 2).

**Table 2:** Laboratory data of studied subjects

	***Cases***	***Control***	***P***
	**(n** **=** **80)**	**(n** **=** **40)**	
**Serum Ca (mg/dL)**	8.5 ± 1.6	10 ±1.4	<0.01^a^
**Serum Po4 (mg/dL)**	5.4 ± 1.5	3.6 ± 0.9	<0.01^a^
**Serum Alkaline phosphatase (IU/L)**	200(44–453)	189(68–451)	> 0.05^b^
**Fasting blood Glucose (mg/dL)**	178(112–316)	94(80–133)	<0.01^b^
**Fasting insulin (uU/mL)**	5.2 ± 1.9	6.7 ± 2.3	< 0.05^a^
**Insulin dose (U/Kg/d)**	0.9 ± 0.1	-	
**HbA1c (%)**	8.6 ± 1.9	-	
**HOMA**-**IR**	3.5 ± 0.7	1.4 ± 0.3	<0.01^a^
**Serum 25 OH Vit D (ng/ml)**	24.7 ± 5.6	26.5 ± 4.8	> 0.05^a^
**Serum PTH (pg/ml)**	57.7 ± 13.2	33.3 ± 8.3	<0.01^a^

HbA1c: glycosylated hemoglobin; HOMA-IR: Homeostasis model for assessment of insulin resistance; Serum PTH: serum parathyroid hormone.

Data are presented as mean ± SD or median (min-max).

*P* value < 0.05 indicates a significant difference.

^a^(*Student t*-*test*). ^b^*Mann*–*Whitney U* test.

A significant difference was observed between vitamin D deficient diabetic cases and those with normal vitamin D level regarding serum calcium, phosphorus, alkaline phosphatase, and serum parathyroid hormone levels (all *P* < 0.01). Moreover, vitamin D deficient diabetic cases had significantly higher HOMA-IR values than those with normal vitamin D level (4.7 ± 0.3 Vs 2.8 ± 0.6; *P* < 0.01). Of note, we observed significantly shorter disease duration in vitamin D deficient diabetic cases compared to those with normal vitamin D level (6.7 ± 2.3 Vs 43 ± 9 months; *P* < 0.01), respectively (Table 3).

**Table 3:** Laboratory data of studied cases according to vitamin D status

	***Vitamin D deficient cases*** **(n** **=** **44)**	***Cases with normal Vitamin D level*** **(n** **=** **36)**	***p***
**Serum Ca (mg/dL)**	7.3 ± 1.0	9.6 ± 1.1	<0.01^a^
**Serum Po4 (mg/dL)**	6.1 ± 1.4	4.4 ± 1.0	<0.01^a^
**Serum alkaline phosphatase (IU/L)**	260 (44–453)	128 (66–251)	<0.01^b^
**Serum PTH (pg/ml)**	66.1 ± 9.4	47.4 ± 9.3	<0.01^a^
**Fasting blood Glucose (mg/dL)>**	208 (115–316)	197 (112–282)	0.05^b^
**Fasting insulin (uU/mL)**	6.7 ± 1.5	7.2 ± 1.2	> 0.05^a^
**Insulin dose (U/Kg/d)**	0.93 ± 0.11	0.91 ± 0.18	> 0.05^a^
**HbA1c (%)**	9.0 ± 1.6	8.4 ± 2.1	> 0.05^a^
**HOMA**-**IR**	.7 ± 0.3	42.8 ± 0.6	<0.01^a^
**Disease duration (months)**	6.7 ± 2.3	43 ± 9	<0.01^a^

Serum PTH: serum parathyroid hormone; HbA1c: glycosylated hemoglobin; HOMA-IR: Homeostasis model for assessment of insulin resistance.

Data are presented as mean ± SD or median (min-max). *P* value < 0.05 indicates a significant difference. ^a^(*Student t*-*test*). ^b^*MannWhitney U* test.

In diabetic cases, serum levels of vitamin D showed significant negative correlations with BMI (*r* = -0.643, *P* < 0.01; Table 4), meanwhile it showed significant positive correlations with disease duration (*r* = 0.544, *P* < 0.01; Table 4) .On the other hand, serum levels of vitamin D did not correlate with clinical parameters, such as age, gender; or with biochemical parameters, such as serum calcium, phosphorus, alkaline phosphatase, fasting blood glucose, HbA1C or HOMA-IR (*all P* > 0.05; Table 4).

**Table 4:** **Correlation between serum vitamin D levels and clinic**-**laboratory parameters in diabetic children**

**Vitamin D vs.**	***r***	***P value***
**Age (years)**	0.213	> 0.05
**Gender**	0.168	> 0.05
**BMI (kg/m**^**2**^**)**	-0.643	<0.01
**Serum Ca (mg/dL)**	0.285	> 0.05
**Serum Po4 (mg/dL)**	-0.196	> 0.05
**Serum Alkaline phosphatase (IU/L)**	-0.113	> 0.05
**Fasting blood Glucose (mg/dL)**	-0.186	> 0.05
**HbA1c (%)**	-0.175	> 0.05
**HOMA-IR**	-0.209	> 0.05
**Disease duration (months)**	0.544	<0.01

BMI: body mass index; HbA1c: glycosylated hemoglobin; HOMA-IR: Homeostasis model for assessment of insulin resistance.

(Pearson’s Correlation analysis).

*P* value < 0.05 indicates a significant difference.

## Discussion

As vitamin D deficiency is considered to be directly involved in inducing immune-mediated β-cell destruction as well as calcium mediated dysfunction leading to onset of clinical Diabetes, the levels of vitamin D must be low at the time of diagnosis. Some previous studies did show that vitamin D levels are low at the time of diagnosis [16,17].

The nationwide Diabetes Incidence Study in Sweden (DISS) found that the 25 OHD levels in the new onset T1D (n = 459) were low compared to controls (*P <* 0*.*001). The vitamin D levels were also low after 8 yr of follow up in T1D [16].

In the present study, we found that serum vitamin D levels were not significantly lower in diabetic subjects compared to the control group (24.7 ± 5.6 vs 26.5 ± 4.8 ng/ml; *P* > 0.05).

These results are different from those of Pozzili et al. who found that the 25(OH) D levels in new onset T1D (n = 88) were low compared to healthy controls (*P <* 0.01). They also studied the correlation between vitamin D and time when diagnosis was made and concluded that vitamin D levels were similarly low during summer and winter months, excluding the possibility of a significant seasonal variation [17].

There are two main biochemical parameters regarding the negative effects of vitamin D deficiency on the skeleton namely ALP and iPTH. The cut-off point of serum 25-OHD in which the mean serum PTH concentration begins to increase is defined as 20 or 30 ng/ml [18].

In our study, we found that serum calcium was significantly lower and both serum phosphorus and serum parathyroid hormone were significantly higher in diabetic subjects compared to control children (all *P* < 0.01).

This agreed with a study performed by Hamed et al who revealed that serum PTH levels were significantly higher in T1DM patients than controls. The explanation of this apparently increased PTH levels might be because of the functioning feedback mechanisms to the decrease in serum levels of calcium and 25(OH) D [19].

By contrast, Kemink et al demonstrated normal or even low PTH concentrations in diabetic patients [20].

Our results showed a significant negative correlation between serum vitamin D and BMI (*r* = -0. 643, *p* < 0.01). This was concordant with the results of the 2001–2004 National Nutrition and Health Survey in the United States indicate that metabolic syndrome prevalence was 3.8 fold higher among obese adolescents whose 25(OH) D levels were lower than 15 ng/mL as compared to those with levels higher than 26 ng/mL [21].

On the other hand, Çizmecioglu et al found that vitamin D deficiency and insufficiency are common in obese and overweight schoolchildren, especially in girls. Obesity could be a risk factor in terms of hypovitaminosis D in adolescents. Vitamin D supplementation should be administered particularly to adolescent girls [22].

Previous cohort studies explained that lower serum 25(OH) D in obese children was most likely the cause of higher iPTH concentrations; and serum iPTH levels were positively correlated with the degree of adiposity and were higher only in the hypovitaminosis D and vitamin D–deficient groups compared with the vitamin D–sufficient subjects [23,24].

In the present study, we found a significant difference between vitamin D deficient cases and those with normal vitamin D level regarding HOMA-IR (4.7 ± 0.3 Vs 2.8 ± 0.6; *P* < 0.01) and diabetes duration (6.7 ± 2.3 Vs 43 ± 9 months; *P* < 0.01), meanwhile no significant difference was observed as regards HbA1c (*P* > 0.05).

This was concordant with Chiu et al who observed a positive relationship between vitamin D status and insulin sensitivity index in adults. In addition, they showed that vitamin D levels were negatively correlated with both first- and second phase insulin responses during a hyperglycemic clamp and glucose levels during oral glucose tolerance test. Thus, they suggested that subjects with hypovitaminosis D not only displayed impaired β-cell function causing impaired glucose homeostasis, but also were at increased risk of developing insulin resistance and metabolic syndrome compared with vitamin D–sufficient subjects [25].

On the other hand, previous studies showed that serum 25(OH) D levels were inversely correlated with HbA1c independent of body fat, implying higher ambient glucose concentrations in children with lower vitamin D concentrations [26,27].

In our study, no significant difference was observed between vitamin D deficient cases and those with normal vitamin D level regarding fasting blood glucose and fasting insulin levels (*P* > 0.05).

These results are concordant with those of a recent report by Erdonmez et al who found that no correlations were found between insulin measurements during oral glucose tolerance test and vitamin D deficiency. They added that mean vitamin D levels were similar in subjects with and without metabolic syndrome (*P* > 0.05) [28].

On the other hand, another study conducted among adolescents of French origin in Canada failed to reveal an association between 25(OH) D level and existence of at least two components of metabolic syndrome. In the same study, it was shown that every 10 ng/mL increment in 25 (OH) D levels causes a mild decrease in the fasting blood glucose levels and HOMA- IR [29].

Our data showed a significant difference between vitamin D deficient cases and those with normal vitamin D level regarding serum calcium, phosphorus, alkaline phosphatase, serum parathyroid hormone levels (all *P* < 0.01); which confirm the results of previous studies [30,31].

Although our data revealed that vitamin D deficiency were more common among diabetic children compared to the control group (*P* < 0.01),our study failed to find a significant difference between diabetic children and healthy controls as regards serum vitamin D levels (*P* > 0.05).

The small sample size was one of our limitations in this study; we suggest that multicenter approaches may be necessary to attain larger sample sizes. Future longitudinal cohort studies are recommended to better investigate the correlations assessed in the present study and to evaluate the effects of time on the analyzed variables. Finally, more extended studies to define the role of vitamin D in the pathogenesis of type 1 diabetes are urgently needed because trials using active form of this metabolite for type 1 diabetes prevention are actively being considered.

## Conclusion

Public health message on the importance of vitamin D status; especially in diabetic children and adolescents, should be disseminated to the public.

## Competing interest

The authors declare that they have no competing interest.

## Authors’ contributions

*SA* participated in the design, collected samples and also participated in the analysis of data and discussion. *SS* participated in the design of the study and performed the statistical analysis. *WE and SMA* reviewed the results and discussion. *AA* participated in the design of the study and helped to draft the manuscript. *AE* conceived of the study and coordinated the sample collection and analysis. All authors read and approved all the manuscript.

## Author information

Department of Pediatrics, Faculty of Medicine, Zagazig University, Egypt. http://www.zu.edu.eg/.
